# Ellipsoid zone reflectivity as a functional imaging biomarker for age-related macular degeneration: a MACUSTAR study report

**DOI:** 10.1038/s41598-025-00735-7

**Published:** 2025-06-20

**Authors:** Yannick N. Liermann, Charlotte Behning, Ben Isselmann, Matthias Schmid, Hannah M. P. Dunbar, Ulrich F. O. Luhmann, Robert P. Finger, Steffen Schmitz-Valckenberg, Frank G. Holz, Maximilian Pfau, Chi D. Luu, Marlene Saßmannshausen, Sarah Thiele, the MACUSTAR consortium, H. Agostini, L. Altay, R. Atia, F. Bandello, P. G. Basile, C. Behning, M. Belmouhand, M. Berger, A. Binns, C. J. F. Boon, M. Böttger, C. Bouchet, J. E. Brazier, T. Butt, C. Carapezzi, J. Carlton, A. Carneiro, A. Charil, R. Coimbra, M. Cozzi, D. P. Crabb, J. Cunha-Vaz, C. Dahlke, L. de Sisternes, H. Dunbar, R. P. Finger, E. Fletcher, H. Floyd, C. Francisco, M. Gutfleisch, R. Hogg, F. G. Holz, C. B. Hoyng, A. Kilani, J. Krätzschmar, L. Kühlewein, M. Larsen, S. Leal, Y. T. E. Lechanteur, M. Liew, U. F. O. Luhmann, A. Lüning, I. Marques, C. Martinho, A. Miliu, G. Montesano, Z. Mulyukov, M. Paques, B. Parodi, M. Parravano, S. Penas, T. Peters, T. Peto, M. Pfau, S. Priglinger, D. Rowen, G. S. Rubin, J. Sahel, C. Sánchez, O. Sander, M. Saßmannshausen, M. Schmid, S. Schmitz-Valckenberg, H. Schrinner-Fenske, J. Siedlecki, R. Silva, A. Skelly, E. Souied, G. Staurenghi, L. Stöhr, D. J. Taylor, J. H. Terheyden, S. Thiele, A. Tufail, M. Varano, L. Vieweg, A. Wolf, N. Zakaria

**Affiliations:** 1 Department of Ophthalmology, University of Bonn, Bonn, Germany; 2 Institute of Medical Biometry, Informatics and Epidemiology, University Hospital Bonn, Bonn, Germany; 3 Institute of Ophthalmology, University College London, London, UK; 4 Department of Optometry, Moorfields Eye Hospital Foundation Trust, London, UK; 5 Roche Pharmaceutical Research and Early Development, Translational Medicine Ophthalmology, Roche Innovation Center Basel, Basel, Schweiz; 6 Department of Ophthalmology, Medical Faculty Mannheim, University of Heidelberg, Mannheim, Germany; 7 John A. Moran Eye Center, University of Utah, Salt Lake City, USA; 8 Institute of Molecular and Clinical Ophthalmology Basel, Basel, Switzerland; 9 Center for Eye Research Australia, Royal Victorian Eye and Ear Hospital, Melbourne, Australia; 10 Ophthalmology, Department of Surgery, The University of Melbourne, Melbourne, Australia; 11 Department of Ophthalmology, University Medical Center Hamburg-Eppendorf, Hamburg, Germany; 12 Novartis Pharma AG, Basel, Switzerland; 13 Centre Hospitalier Intercommunal de Creteil (HIC), University Eye Clinic, Centre Hospitalier Creteil, Paris, France; 14 STZ Biomed & STZ Eyetrial at the Center of Ophthalmology, University Hospital Tuebingen, Tuebingen, Germany; 15 Ludwig-Maximilians-Universitaet Muenchen (LMU), University Eye Hospital Munich, Munich, Germany; 16 G. B. Bietti Eye Foundation-IRCCS, Rome, Italy; 17 Department of Ophthalmology, Universitaetsklinikum Freiburg (UKLFR), University of Freiburg, Freiburg, Germany; 18 Department of Ophthalmology Rigshospitalet-Glostrup, Copenhagen University Glostrup, Copenhagen, Denmark; 19 Department of Ophthalmology Luigi Sacco Hospital, University of Milan, Milan, Italy; 20 Ophthalmology and Vision Sciences The Queen’s University an Royal Group of Hospitals Trust Belfast, Belfast, Northern Ireland; 21 Department of Ophthalmology, University of Ulm, Ulm, Germany; 22 Clinical Trial Unit, Department of Ophthalmology Gloucestershire Hospitals, NHS Foundation Trust Cheltenham, Cheltenham, UK; 23 Department of Ophthalmology, University Vita Salute-Scientific Institute of San Raffael, Milan, Italy; 24 Universität zu Köln, Zentrum für Augenheilkunde, Cologne, Germany; 25 Department of Ophthalmology, Leiden University Medical Center, Leiden, The Netherlands; 26 Department of Ophthalmology, St. Franziskus Hospital, Münster, Germany; 27 BAYER AG, Leverkusen, Germany; 28 F. Hoffmann-La Roche Ltd, Basel, Switzerland; 29 European Clinical Research Infrastructure Network (ECRIN), Paris, France; 30 AIBILI Associaton for Innovation and Biomedical Research on Light and Image (AIBILI), Coimbra, Portugal; 31 Department of Ophthalmology Porto Medical School, Centro Hospitalar de Sao Joao EPE (Hospital Sao Joao) Hospital S. Joao Porto, Porto, Portugal; 32 Centre Hospitalier National d’Opthalmologie des Quinze-Vingts, Paris, France; 33 Moorfields Eye Hospital NHS Foundation Trust (MBRC), London, UK; 34 Stichting Katholieke Universiteit/Radboud University Medical Center (SRUMC), Radbound University, Nijmegen Medical Center, Nijmegen, The Netherlands; 35 City University London, London, UK; 36 Fondation Voir et Etendre, Paris, France; 37 University of Sheffield, Sheffield, UK; 38 University College London (UCL), London, UK; 39 Carl Zeiss Meditec, AG., Jena, Germany; 40 Institute for Medical Biometry, Informatics and Epidemiology, Medical Faculty, University of Bonn, Bonn, Germany

**Keywords:** Diagnostic markers, Macular degeneration

## Abstract

This study evaluated the functional relevance of relative ellipsoid zone reflectivity (rEZR) on spectral-domain optical coherence tomography as a structural biomarker for retinal integrity, focusing on its association with retinal function. Participants with age-related macular degeneration (AMD) and controls from the MACUSTAR study underwent functional testing, including mesopic fundus-controlled perimetry, best-corrected visual acuity, low-luminance visual acuity, low-luminance deficit, Moorfields Acuity Test, and Pelli-Robson contrast sensitivity, along with spectral-domain optical coherence tomography imaging. Structural and functional data were analyzed globally and spatially aligned for topographic analysis. Linear-mixed effects models, adjusted for age, sex, and eccentricity of the rEZR, assessed associations between rEZR and functional metrics. A total of 275 eyes (early AMD, n = 34; intermediate AMD, n = 152; late AMD, n = 36; controls, n = 53) from 275 participants (mean ± standard deviation age: 71.1 ± 7.2 years; 63.3% female) were included. In global analyses, rEZR was associated with the mean average threshold in mesopic fundus-controlled perimetry (coefficient estimate 0.0492, 95% confidence interval 0.0190–0.0794, p = 0.0015), low-luminance visual acuity (coefficient estimate − 0.0015, 95% confidence interval − 0.0026 to − 0.0004, p = 0.0092), Moorfields Acuity Test (coefficient estimate 0.0092, 95% confidence interval − 0.0022 to − 0.0001, p = 0.0285), and Pelli-Robson contrast sensitivity (coefficient estimate 0.0030, 95% confidence interval 0.0015–0.0045, p = 0.0001). Topographic analysis further revealed an association of rEZR with mesopic retinal sensitivity (coefficient estimate 0.0065, 95% confidence interval 0.0026–0.0104, p < 0.0001). Higher outer retinal reflectivity is linked to better retinal function in AMD and controls, supporting its potential as a biomarker for retinal integrity and function.

## Introduction

Age-related macular degeneration (AMD) is a common cause of central visual impairment in the aging population worldwide, significantly affecting quality of life^1,2^. It manifests as a chronic-progressive disease, in which drusen and pigmentary abnormalities characterize the intermediate and geographic atrophy (GA) and/or macular neovascularization (MNV) advanced stage^3,4^. Since visual loss predominantly occurs in advanced AMD, therapeutic interventions targeting the intermediate stage (iAMD) are of considerable interest^5^. Yet, the challenge of identifying sensitive and reliable biomarkers for iAMD that could serve as valid outcome measures in the context of upcoming interventional clinical trials remains.

On spectral-domain optical coherence tomography (SD-OCT) the relative ellipsoid zone reflectivity (rEZR), comprising the reflectivity signal of both the ellipsoid zone (EZ) and the external limiting membrane (ELM), has emerged as a promising quantitative measure for assessing outer retinal integrity^6–10^. The FDA has recently approved the assessment of the EZ integrity as a structural endpoint in GA trials, acknowledging its importance for photoreceptor degeneration in AMD^11^. However, in earlier stages of AMD, complete EZ loss is uncommon, making reflectivity a more sensitive measure than integrity alone. Previous studies employing an automated approach for rEZR determination have demonstrated a longitudinal rEZR decline in AMD patients as well as its association with AMD staging and the presence of high-risk features in iAMD^6,12–15^. Given the presumed origin of the EZ signal—i.e., mitochondria within photoreceptor inner segments—and the ELM as a linear confluence of junctional complexes supporting the photoreceptors, it can be hypothesized that structurally assessed rEZR might also reflect outer retinal function^16–22^. However, the functional relevance of rEZR is yet unclear. Its validation against established measures of retinal function is required to better understand its clinical relevance.

This study, conducted as part of the MACUSTAR study cross-sectional baseline cohort, assesses the association between rEZR and multiple measures of retinal function across various stages of AMD, both globally and topographically. By analyzing the rEZR’s potential as a structural surrogate for retinal function (“functional retinal imaging”), this research addresses the critical need for innovative biomarker identification that could be accepted by regulatory agencies as a novel clinical endpoint for future iAMD trials.

## Results

### Group characteristics

A total of 275 eyes from 275 study participants (female: n = 174; 63.3%) with a mean age of 71.1 ± 7.2 years were included in the analysis. According to the AMD disease staging 34 (12.4%) participants were categorized as early AMD, 152 (55.3%) as iAMD and 36 (13.1%) as late-stage AMD (26 with GA, 10 with MNV). Additionally, 53 (19.3%) individuals of the control subgroup were included.

The mean global rEZR for the overall study population was 36.9 ± 18.9 AU. Specifically, the early AMD group exhibited a rEZR of 41.2 ± 17.3 AU, the intermediate AMD group 36.9 ± 16.7 AU, and the late AMD group 16.3 ± 10.9 AU. In contrast, healthy controls demonstrated a higher rEZR of 47.8 ± 19.2 AU.

Assessment of retinal function using fundus-controlled perimetry (FCP) revealed a mean mesopic average threshold of 21.8 ± 6.8 dB across the entire study population, with subgroup means of 23.9 ± 2.6 dB in early AMD, 23.3 ± 4.0 dB in intermediate AMD, 8.0 ± 6.9 dB in late-stage AMD, and 25.4 ± 2.0 dB in the control group. Chart-based visual function testing showed a mean best-corrected visual acuity (BCVA) of 0.10 ± 0.29 logMAR [80.0 ± 14.5 ETDRS letters], mean low-luminance visual acuity of 0.302 ± 0.291 logMAR [69.9 ± 14.5 ETDRS letters], mean low-luminance deficit of − 0.196 ± 0.127 logMAR [94.8 ± 6.3 ETDRS letters], mean Moorfields Acuity Test of 0.498 ± 0.252 logMAR [60.1 ± 12.6 ETDRS letters], and mean Pelli-Robson contrast sensitivity of 1.53 ± 0.272 logCS [33.6 ± 5.4 ETDRS letters] for the entire study population. For further subgroup results and baseline characteristics, please see Table 1.

**Table 1:** Baseline characteristics of the study group. *AMD* Age-related macular degeneration, *iAMD* Intermediate age-related macular degeneration, *rEZR* Relative ellipsoid zone reflectivity, *AU* Arbitrary units, *FCP* Fundus-controlled perimetry, *dB* Decibels, *BCVA* Best-corrected visual acuity, *logMAR* Logarithm of the minimum angle of resolution, *LLVA* Low-luminance visual acuity, *LLD* Low-luminance deficit, *MAT* Moorfields Acuity Test, *PR* Pelli-Robson contrast sensitivity, *logCS* Logarithm of contrast sensitivity.

Variable	No AMD (n = 53)	Early AMD (n = 34)	iAMD (n = 152)	Late AMD (n = 36)	Overall (n = 275)
Age (years)					
Mean (SD)	68.1 (6.40)	71.7 (6.38)	71.2 (7.61)	74.6 (5.69)	71.1 (7.23)
Median (min., max.)	68.0 [55.0, 80.0]	72.0 [57.0, 82.0]	72.0 [55.0, 88.0]	74.0 [64.0, 84.0]	72.0 [55.0, 88.0]
Sex (male)					
n (%)	22 (41.5%)	7 (20.6%)	54 (35.5%)	18 (50.0%)	101 (36.7%)
Central subfield rEZR (AU)					
Mean (SD)	50.3 (21.2)	36.6 (15.2)	25.6 (13.9)	5.24 (7.92)	29.9 (20.0)
Median (min., max.)	54.3 [9.73, 101]	38.0 [6.78, 75.0]	23.5 [1.44, 70.0]	3.23 [1.03, 43.7]	26.9 [1.03, 101]
Global rEZR (AU)					
Mean (SD)	47.8 (19.2)	41.2 (17.3)	36.9 (16.7)	16.3 (10.9)	36.9 (18.9)
Median [min., max.]	41.3 [11.6, 101]	35.7 [16.3, 96.8]	34.4 [2.28, 84.7]	12.0 [4.52, 50.5]	34.3 [2.28, 101]
Mesopic sensitivity (dB)					
Mean (SD)	25.4 (2.05)	23.9 (2.61)	23.3 (4.02)	8.03 (6.91)	21.8 (6.77)
Median [min., max.]	25.7 [19.4, 29.2]	24.6 [17.1, 27.6]	24.2 [0.500, 28.8]	7.50 [0, 21.1]	24.2 [0, 29.2]
BCVA (logMAR)					
Mean (SD)	− 0.0404 (0.0838)	0.0106 (0.0834)	0.0238 (0.105)	0.763 (0.250)	0.107 (0.286)
Median [min., max.]	− 0.0600 [− 0.240, 0.140]	0.0200 [− 0.180, 0.200]	0.0200 [− 0.240, 0.280]	0.840 [0.200, 1.10]	0.0200 [− 0.240, 1.10]
LLVA (logMAR)					
Mean (SD)	0.137 (0.0903)	0.188(0.142)	0.237(0.151)	0.929 (0.236)	0.302(0.291)
Median [min., max.]	0.120[− 0.0200, 0.380]	0.170 [− 0.0400, 0.500]	0.220 [− 0.140, 0.680]	0.940 [0.520, 1.52]	0.200 [− 0.140, 1.52]
MAT (logMAR)					
Mean (SD)	0.353 (0.103)	0.418 (0.118)	0.441 (0.143)	1.03 (0.197)	0.498 (0.252)
Median [min., max.]	0.340 [0.160, 0.620]	0.410 [0.200, 0.720]	0.420 [0.100, 0.900]	1.00 [0.660, 1.46]	0.420 [0.100, 1.46]
PR (logCS)					
Mean (SD)	1.71 (0.165)	1.63 (0.156)	1.56 (0.177)	1.09 (0.346)	1.53 (0.272)
Median [min., max.]	1.75 [1.05, 1.95]	1.65 [1.25, 1.90]	1.55 [1.05, 1.95]	1.20 [0.200, 1.55]	1.60 [0.200, 1.95]
LLD (logMAR)					
Mean (SD)	− 0.177 (0.0660)	− 0.178 (0.104)	− 0.213 (0.103)	− 0.166 (0.249)	− 0.196 (0.127)
Median [min., max.]	− 0.180 [− 0.320, − 0.0200]	− 0.170 [− 0.420, 0.0200]	− 0.200 [− 0.640, − 0.0200]	− 0.110 [− 0.820, 0.400]	− 0.180 [− 0.820, 0.400]

### Association between rEZR and FCP-derived retinal sensitivity

**Fig. 1:**
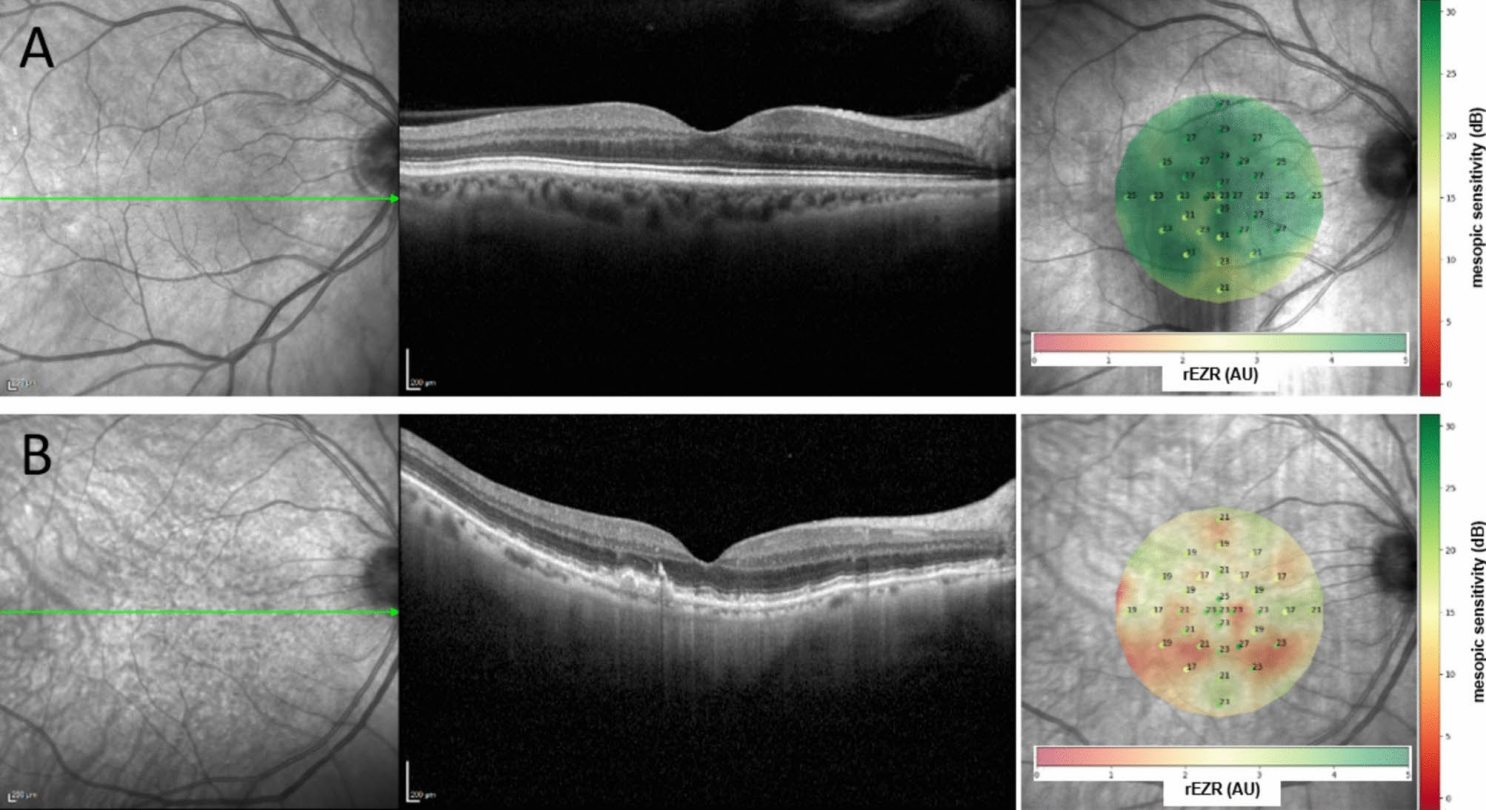
Exemplary cases of an healthy individual (**A**) and a participant with intermediate age-related macular degeneration (**B**) with (from left to right) confocal near-infrared en-face imaging, the horizontal OCT line through the fovea and a heat-map representation of the spatial association of the relative ellipsoid zone reflectivity (rEZR) and retinal sensitivity as tested by mesopic fundus-controlled perimetry (FCP). In the heat-map representation, the rEZR (AU) is represented in the background with lower and higher values ranging from red to green, while point-wise retinal sensitivity is demonstrated by superimposed specific values (dB) and also color-coded stimulus points, again with lower values in red and higher values in green. Note the association of lower rEZR values (more red-colored areas) with worse retinal function, as well as higher rEZR values (more green-colored areas) with better retinal function.

We found a significant univariate association between rEZR and FCP-derived retinal sensitivity in global (coefficient estimate (CE) 0.1787 [95%-CI 0.1416–0.2159, p < 0.0001]), locally averaged (CE 0.1164 [95%-CI 0.0922–0.1406, p < 0.0001]), and spatially resolved (CE 0.0076 [95%-CI 0.0031–0.0122, p < 0.0001]) analyses, with higher rEZR values corresponding to better retinal function. This association remained significant after adjustment for age, sex and AMD staging, given a CE of 0.0492 (95%-CI 0.0190–0.0794, p = 0.0015) AU in global analysis, 0.0247 (95%-CI 0.0039–0.0455, p = 0.0200) AU in locally averaged analysis and 0.0065 (95%-CI 0.0026–0.0104, p = 0.0010) AU in the spatially resolved analysis, respectively. For graphical representation of the results in representative cases from the iAMD and control subgroups, please refer to Fig. 1. For detailed results of the adjusted and univariate models, see Table 2 and Supplementary Table 1, respectively.

**Table 2:** Association of the relative Ellipsoid Zone Reflectivity (rEZR) and the mesopic average threshold (mesAT [dB]) as assessed in fundus-controlled perimetry (FCP) in global, locally averaged and spatially resolved analysis. Multivariable models were fitted with FCP as outcome variable. Independent variables included mean rEZR (in AU), AMD stage as a categorical variable with no AMD group as a reference category, age in years and sex with female sex as reference.

	Predictors	mesAT [dB]		
		Coefficient estimate	95%-Confidence interval	p-value
Global	(Intercept)	29.5041	23.8335 to 35.1747	< 0.0001
	Mean rEZR [AU]	0.0492	0.0190 to 0.0794	0.0015
	AMD stage [early]	− 0.9677	− 2.7009 to 0.7655	0.2726
	AMD stage [intermediate]	− 1.3611	− 2.6373 to − 0.0849	0.0367
	AMD stage [late]	− 15.2023	− 17.0957 to − 13.3088	< 0.0001
	Age [years]	− 0.0906	− 0.1627 to − 0.0184	0.0141
	Sex [male]	− 0.6307	− 1.6161 to 0.3547	0.2087
Locally averaged	(Intercept)	31.3301	25.8129 to 36.8473	< 0.0001
	Mean rEZR [AU]	0.0247	0.0039 to 0.0455	0.0200
	AMD stage [early]	− 0.9375	− 2.6906 to 0.8156	0.2933
	AMD stage [intermediate]	− 1.2205	− 2.5633 to 0.1223	0.0747
	AMD stage [late]	− 15.2388	− 17.2655 to − 13.2121	< 0.0001
	Age [years]	− 0.1066	− 0.1780 to − 0.0352	0.0036
	Sex [male]	− 0.6807	− 1.6763 to 0.3149	0.1794
Spatially resolved	(Intercept)	34.7798	29.9200 to 39.6397	< 0.0001
	Mean rEZR [AU]	0.0053	0.0008 to 0.0098	0.0213
	AMD stage [early]	− 1.115	− 2.8956 to 0.6727	0.2221
	AMD stage [intermediate]	− 1.5605	− 2.8571 to − 0.2640	0.0183
	AMD stage [late]	− 16.2982	− 18.1017 to − 14.4947	< 0.0001
	Age [years]	− 0.1303	− 0.2000 to − 0.0607	0.0002
	Sex [male]	− 0.7716	− 1.7889 to 0.2457	0.1371

### Association between rEZR and other chart-based visual function tests

In multivariable analyses, rEZR was associated with low-luminance visual acuity, Moorfields Acuity Test, and Pelli-Robson contrast sensitivity in the global analysis, with coefficient estimates of − 0.0015 (95%-CI − 0.0026 to − 0.0004, p = 0.0092), − 0.0011 (95%-CI − 0.0022 to − 0.0001, p = 0.0285), and 0.0030 (95%-CI 0.0015–0.0045, p = 0.0001), respectively. No association (p = 0.2572) was found between rEZR and BCVA (CE − 0.0006; 95%-CI − 0.0015 to 0.0004). Similarly, no association was observed between rEZR and low-luminance deficit in the global analysis (CE 0.0010; 95%-CI − 0.0000 to 0.0019, p = 0.0521).

In the central subfield analysis (ETDRS grid, central 1 mm diameter), the rEZR was significantly associated with low-luminance visual acuity and Pelli-Robson contrast sensitivity given CEs of − 0.0013 (95%-CI − 0.0025 to 0.001; p = 0.0306) and 0.0019 (95%-CI 0.004 to 0.0035; p = 0.0128). Additionally, rEZR showed a significant association with low-luminance deficit in the central subfield (CE: 0.0011; 95%-CI 0.0001–0.0021, p = 0.0343). Neither BCVA (CE: − 0.0002; 95%-CI − 0.0012 to 0.0008; p = 0.6541) nor Moorfields Acuity Test (CE: − 0.0007; 95%-CI − 0.0017–0.0004; p = 0.2031) showed association with the mean rEZR of the central ETDRS subfield.

For detailed model results of global and spatially resolved analysis of the association between rEZR and chart-based visual function, please see Table 3. For the corresponding univariate models, refer to Supplementary Table 2.

**Table 3:** Association of relative ellipsoid zone reflectivity (rEZR) with functional tests, including best-corrected visual acuity (BCVA), low-luminance visual acuity (LLVA), Moorfields Acuity Test (MAT), Pelli-Robson contrast sensitivity (PR), and low-luminance deficit (LLD), in global and central-subfield analyses. Multivariable models were fitted with functional tests as the outcome variable. Independent variables included mean rEZR (arbitrary units, AU), AMD stage as a categorical variable with the no AMD group as the reference category, age in years, and sex with female sex as the reference category.

	Predictors	BCVA [logMAR]			LLVA [logMAR]			MAT [logMAR]			PR [logCS]			LLD [logMAR]		
		Coefficient estimate	95%-Confidence interval	p-value	Coefficient estimate	95%-Confidence interval	p-value	Coefficient estimate	95%-Confidence interval	p-value	Coefficient estimate	95%-Confidence interval	p-value	Coefficient estimate	95%-Confidence interval	p-value
Global	(Intercept)	− 0.2756	− 0.4556 to − 0.0957	0.0028	− 0.1465	− 0.3589 to 0.0658	0.1755	0.0202	− 0.1722 to 0.2125	0.8367	1.7991	1.5188 to 2.0794	< 0.0001	− 0.1291	− 0.3101 to 0.0519	0.1613
	Mean rEZR [AU]	− 0.0006	− 0.0015 to 0.0004	0.2572	− 0.0015	− 0.0026 to − 0.0004	0.0092	− 0.0011	− 0.0022 to − 0.0001	0.0285	0.0030	0.0015 to 0.0045	0.0001	0.0010	− 0.000 to 0.0019	0.0521
	AMD stage [early]	0.0293	− 0.0257 to 0.0843	0.2954	0.0212	− 0.0437 to 0.0861	0.5213	0.0301	− 0.0287 to 0.0889	0.3136	− 0.0438	− 0.1294 to 0.0419	0.3155	0.0081	− 0.0472 to 0.0634	0.7730
	AMD stage [intermediate]	0.0448	0.0043 to 0.0853	0.0304	0.0669	0.0191 to 0.1147	0.0063	0.0557	0.0124 to 0.0990	0.0119	− 0.1071	− 0.1702 – − 0.0441	0.0009	− 0.0221	− 0.0628 to 0.0186	0.2866
	AMD stage [late]	0.7612	0.7011 to 0.8213	< 0.0001	0.7102	0.6393 to 0.7811	< 0.0001	0.6022	0.5380 to 0.6665	< 0.001	− 0.5043	− 0.5979 – − 0.4107	< 0.0001	0.0510	− 0.0094 to 0.1114	0.0979
	Age [years]	0.0040	0.0017 to 0.0062	0.0008	0.0053	0.0026 to 0.0080	0.0002	0.0059	0.0034 to 0.0083	< 0.0001	− 0.0035	− 0.0071 to 0.0000	0.0530	− 0.0013	− 0.0036 to 0.0010	0.2664
	Sex [male]	− 0.173	− 0.0485 to 0.0140	0.2777	− 0.0046	− 0.0415 to 0.0323	0.8072	− 0.0282	− 0.0616 to 0.0053	0.0983	0.0127	− 0.0360 to 0.0614	0.6087	− 0.0127	− 0.0442 to 0.0188	0.4274
Spatially resolved	(Intercept)	− 0.3141	− 0.4775 to − 0.1506	0.0002	− 0.1883	− 0.3825 to 0.0060	0.0575	− 0.0514	− 0.2271 to 0.1243	0.5650	2.0098	1.7578 to 2.2617	< 0.0001	− 0.1258	− 0.2899 to 0.0383	0.1324
	Mean rEZR [AU]	− 0.0002	− 0.0012 to 0.0008	0.6541	− 0.0013	− 0.0025 to 0.001	0.0306	− 0.0007	− 0.0017 to 0.0004	0.2031	0.0019	0.004 to 0.0035	0.0128	0.0011	0.0001 to 0.0021	0.0343
	AMD stage [early]	0.0271	− 0.0279 to 0.0822	0.3330	0.0107	− 0.0547 to 0.0762	0.7468	0.0255	− 0.0336 to 0.0847	0.3961	− 0.0300	− 0.1148 to 0.0549	0.4873	0.0164	− 0.0389 to 0.0716	0.5600
	AMD stage [intermediate]	0.0406	− 0.0049 to 0.0861	0.0801	0.0430	− 0.011 to 0.0971	0.1184	0.0459	− 0.0030 to 0.0947	0.0657	− 0.0825	− 0.1526 to − 0.0124	0.0213	− 0.0024	− 0.0481 to 0.0432	0.9169
	AMD stage [late]	0.7352	0.6636 to 0.8068	< 0.0001	0.7013	0.6162 to 0.7864	< 0.0001	0.5851	0.5082 to 0.6621	< 0.0001	− 0.4793	− 0.5896 to − 0.3689	< 0.0001	0.0339	− 0.0380 to 0.1058	0.3538
	Age [years]	0.0043	0.0022 to 0.0065	0.0001	0.0058	0.0032 to 0.0083	< 0.0001	0.0066	0.0043 to 0.0089	< 0.0001	− 0.0059	− 0.0092 to − 0.0026	0.0005	− 0.0015	− 0.0036 to 0.1058	0.3538
	Sex [male]	− 0.0237	− 0.0553 to 0.0079	0.1413	− 0.0078	− 0.0454 to 0.0298	0.6831	− 0.0281	− 0.0621 to 0.0059	0.1044	0.0050	− 0.0437 to 0.0538	0.8387	− 0.0159	− 0.0476 to 0.0158	0.3251

## Discussion

In this study we found significant associations between the rEZR and a comprehensive battery of functional outcomes demonstrating the functional relevance of the rEZR. Thus, the rEZR shows not only potential as a novel quantitative measure for outer retinal integrity, but also as a structural indicator for retinal function in AMD (“functional imaging”).

The EZ signal in SD-OCT imaging is assumed to originate from mitochondria within photoreceptors, which are essential for metabolism and health, and exhibit light-scattering properties due to their optical reflectivity^18,24,25^. This reflectivity, impacted by photoreceptor function and integrity, indicates that changes in the EZ signal can reflect compromised photoreceptor function^9^. Additionally, the ELM represents the junctional complex between photoreceptors and Müller glial cells, crucial for outer retinal health^26^. Therefore, based on these relationships, analyzing rEZR as a potential surrogate for retinal function is biologically plausible.

While patients with advanced AMD stages experience significant reduction in high-contrast, high-luminance BCVA, patients with earlier stages, specifically iAMD patients, suffer from functional impairment beyond BCVA assessment, presenting with prolonged dark adaptation, reduced contrast sensitivity, localized deficits of FCP-derived retinal sensitivity and difficulties with vision under dim light conditions^27–29^.

Our results demonstrated significant associations between rEZR and these specific functional tests, highlighting the utility of rEZR as a potential biomarker for iAMD. The strong association between rEZR and low-luminance visual acuity, Pelli-Robson contrast sensitivity, and FCP underscores the relevance of the rEZR in reflecting the retinal changes that affect critical visual functions in AMD. With regard to mesopic FCP-testing, higher outer retinal reflectivity (indicated by higher rEZR values) showed significant association (p = 0.0015) with better retinal function given a CE of 0.0492 (95%-CI 0.0190–0.0794) AU in the global analysis. This applied also for rEZR’s association with low-luminance visual acuity and Pelli-Robson contrast sensitivity. In contrast, no association of rEZR was found for BCVA in the global model (CE of − 0.0006 [95%-CI − 0.0015 to 0.0004]; p = 0.2572), nor in the central subfield model (CE of − 0.0002 [95%-CI − 0.0012 to 0.0008]; p = 0.6541). This might indicate that rEZR reflects functional changes in AMD beyond BCVA assessment alone^39^.

Beyond rEZR, several OCT-derived metrics related to the EZ have been previously studied as biomarkers of photoreceptor integrity and function. EZ integrity, typically assessed based on its continuity or disruption, has been correlated with visual acuity and retinal sensitivity in AMD as well as in other retinal diseases, including Best vitelliform macular dystrophy, macular telangiectasia, and retinal vein occlusions^20^. In AMD, greater EZ disruption has been associated with worsening visual function, with higher EZ integrity—including less EZ attenuation, greater EZ-RPE thickness, and higher EZ intensity—correlating with better visual acuity. Additionally, baseline EZ integrity metrics were predictive of future visual acuity loss, further supporting their prognostic value in disease progression^30^. Furthermore, variations in outer retinal substructure thickness, including the photoreceptor inner and outer segments, have been correlated with visual acuity in dry AMD, reinforcing the relevance of these parameters in assessing disease severity^31^. Recently, Birner et al. demonstrated a significant association between ellipsoid zone thickness and loss, as quantified by deep learning algorithms, and retinal sensitivity assessed by microperimetry in geographic atrophy, further emphasizing the functional relevance of EZ-related OCT metrics^32^. While these established EZ metrics provide valuable structural information, they primarily reflect later stages of photoreceptor damage. In contrast, the rEZR offers a quantitative assessment of EZ reflectivity that may detect subtle functional impairments before visible morphological changes occur. Its normalization has been proposed to improve reproducibility across imaging conditions. The strong associations observed between rEZR and functional measures in this study suggest that the rEZR may complement existing EZ biomarkers.

Another important finding of this study is the significant association of rEZR not only on an eye-level (global analysis), but also on a more granular, i.e. spatially resolved level. Particularly in the context of the presented FCP analysis, where both rEZR and retinal function were determined and evaluated in a spatially resolved manner, our results underscore the rEZR’s potential to reflect localized functional deficits. Specifically, we found CEs of 0.0246 (95%-CI 0.0039–0.0455) AU in the locally averaged analysis (p = 0.02) and 0.0065 (95%-CI 0.0026–0.0104) AU in the spatially resolved analysis (p = 0.001). These results support the rEZR’s potential as an innovative outcome measure in future iAMD trials, as its ability to identify retinal areas with functional deficits could have significant implications for targeted therapeutic interventions.

While the relationship between the rEZR and AMD stage, as well as various structural high-risk features associated with iAMD, has been previously investigated, this study is, to the best of our knowledge, the first to assess the spatially resolved functional relevance of quantitative EZ reflectivity changes in AMD^12^. Previously, Wu et al. demonstrated an association between relative EZ intensity—formerly referred to as the inner segment band—and retinal function, assessed by multifocal electroretinography, in patients with AMD exhibiting large drusen^33^. However, unlike our study, the EZ intensity measurements were performed manually on a single OCT line scan, and the spatial resolution of multifocal electroretinography testing was limited. Several other studies have demonstrated the functional relevance of EZ metrics, supporting our findings, although most did not employ spatially resolved analysis^30,34–41^. Yordi et al. demonstrated that longitudinal changes in EZ integrity were significantly associated with visual acuity outcomes in neovascular AMD, particularly emphasizing the role of subretinal hyperreflective material and EZ integrity in predicting visual outcomes^30^. Similarly, Wu et al. concluded in 2014 that the integrity of the ISe band might serve as a surrogate marker of retinal function based on its prognostic value for predicting microperimetric retinal sensitivity^39^. Assessing dark adaptation in AMD subjects, Laíns et al. also highlighted the relevance of the EZ, albeit only having determined its integrity qualitatively, with impaired retinal function given delayed rod-intercept times (RIT) in the presence of EZ disruption^42^.

Several limitations need to be considered in this study. First, as a cross-sectional study analysis, it does not allow for the assessment of rEZR’s prognostic value for future functional impairment and vision loss. Additionally, while this is the first analysis of rEZR and its functional relevance in AMD, it includes the entire MACUSTAR cohort of the cross-sectional study part at baseline, encompassing subjects with different AMD stages and healthy controls. While this enhances the generalizability of our results, a more refined analysis focusing on AMD subgroups as well as longitudinal analyses, are warranted as both functional and phenotypic variations exist particularly with regard to iAMD. Although this study did not directly compare rEZR with traditional AMD biomarkers such as drusen area or retinal layer thickness, prior research has demonstrated its complementary role^12^. Notably, visual function may be impaired even in areas without structural changes, as shown in previous studies, suggesting that the rEZR captures functional deficits beyond traditional biomarkers^43^. Although calculating the rEZR as a ratio reduces variability from scan intensity and optical media opacities, subtle effects from differences in illumination, focal lens or vitreous opacities remain possible.

A major strength of this study is the prospectively and highly standardized acquired retinal imaging and functional data from a multicenter trial and the use of innovative data processing approaches. Specifically, the application of the deep learning-based algorithm “SuperRetina” enabled precise alignment of structural and functional study data, ensuring reliable interpretation of the spatially resolved analyses^32^.

In conclusion, this study demonstrates the functional relevance of outer retinal reflectivity changes, specifically the rEZR, in individuals with AMD and healthy subjects. It highlights the value of the rEZR as an easily acquired and objectively determined biomarker for assessing outer retinal and functional impairment. Further analyses are needed to better understand the clinical implications of the rEZR, particularly its prognostic relevance for progression and visual impairment in patients with iAMD. This will be helpful for patient selection and efficacy assessments in future interventional clinical iAMD trials.

## Methods

### MACUSTAR study participants

The MACUSTAR study (ClinicalTrials.gov Identifier: NCT03349801), a prospective multicenter, low-intervention natural history study, aims to identify novel biomarkers for iAMD^44^. The study’s design and participant selection criteria have been outlined in prior publications^44–46^. Enrolment occurred from March 2018 to February 2020, selecting one eye per participant for the study, prioritizing the eye with better visual acuity when both eyes met the inclusion criteria. In accordance with the Declaration of Helsinki, ethical guidelines were strictly followed, with informed written consent obtained from all participants. The study includes four groups: early AMD, iAMD, late-stage AMD, and a control group^3^. Consistent with the classification system proposed by Ferris et al., iAMD was defined by the presence of large sub-RPE drusen (> 125 µm) and/or any AMD-related pigmentary abnormalities in both eyes^3^. If the fellow eye had an extrafoveal GA lesion, it was required to be no larger than 1.25 mm^2^. Early AMD was characterized by medium-sized drusen (63–125 µm), while late-stage AMD included MNV and/or central GA cases. All image grading and classification of study eyes were performed centrally at the GRADE Reading Center Bonn by trained and independent graders, following the MACUSTAR standardized grading protocol^44^.

In total 301 participants were recruited and included in the cross-sectional part of the MACUSTAR study (early AMD n = 34, iAMD n = 168, late AMD n = 43, controls n = 56). Out of those, 26 participants were not assessed in this study due to incomplete functional data (no AMD n = 2, iAMD n = 8, late AMD n = 6) or missing determination of the rEZR at positions of the MAIA stimulus grid (no AMD n = 1, iAMD n = 8, late AMD n = 1). Reasons for missing microperimetry assessments were mostly procedural errors, e.g. incorrect grid use or incomplete upload of data.

### Imaging protocol

Participants underwent multimodal retinal imaging following standardized operating procedures conducted by certified study site personnel. Prior to imaging, pupil dilation was achieved using tropicamide 0.5% and phenylephrine 2.5% eye drops. Retinal imaging included combined confocal scanning laser ophthalmoscopy for near-infrared reflectance imaging [Automated Real-Time mode (ART) ≥ 30 single frames] and SD-OCT [30° × 25°, enhanced-depth-imaging, high-speed mode, 241 B-scans, distance 30 µm, ART mode = 9] which was acquired using the Spectralis HRA + OCT device (Heidelberg Engineering, Heidelberg, Germany).

### Determination of the relative EZ reflectivity

The rEZR was calculated as the ratio of the peak reflectivity of the EZ to the peak reflectivity of the ELM, using raw OCT images to ensure precise analysis of native, untransformed reflectivity signals (dynamic range: 0 to 1 [arbitrary units, AU]). This ratio minimizes the impact of acquisition-related variability or noise, including potential differences in illumination or scan intensity, as such effects are expected to influence both layers similarly, effectively canceling out when expressed as a ratio^12,47^. The ELM was selected as the reference layer due to its well-documented stability across a wide range of retinal eccentricities, including the fovea, as reported in previous studies^6,48^. Furthermore, as a non-neural structure, the ELM is less susceptible to reflectivity changes associated with aging or early retinal degeneration, making it a reliable benchmark for rEZR determination.

**Fig. 2:**
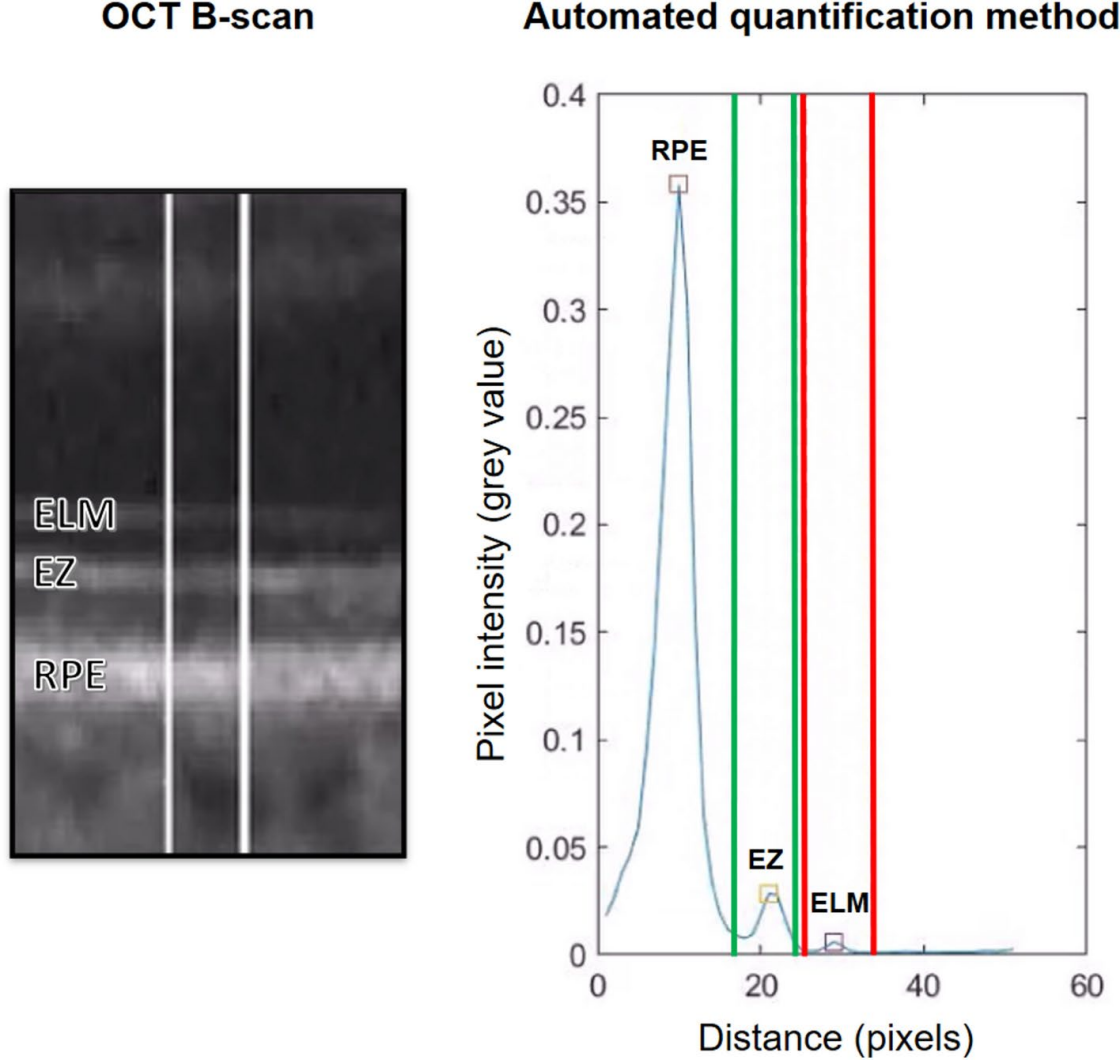
A representative case illustrating peak determination by the automated method. The left panel shows an OCT B-scan with the external limiting membrane (ELM), ellipsoid zone (EZ), and retinal pigment epithelium (RPE) labeled. White lines indicate the region of interest used for reflectivity analysis. The right panel presents the corresponding pixel intensity profile, where green and red vertical lines mark the peak detection areas for EZ and ELM, respectively. Colored rectangles denote peaks identified by the algorithm: RPE (red), EZ (yellow), and ELM (purple).

An automated algorithm (Python Software Foundation, Python Language Reference, version 3.9. Available at [http://www.python.org]; annotated code available at: https://github.com/ bisselma/relEZIquantification) was employed to calculate the rEZR, as previously validated and described in detail^15,47,49,50^. Briefly, segmentation coordinates, obtained via a deep learning-based approach, were superimposed on the raw, non-logarithmic OCT images and used to straighten each B-scan along the RPE^51^. This alignment ensured accurate rEZR calculation, even in eyes with pronounced posterior pole curvature. Regions of interest were defined at adjoining nine-pixel intervals along the x-axis of each B-scan. Within these regions of interest, reflectivity profiles were generated and the EZ and ELM peak reflectivities were automatically identified. Predefined subregions, based on the 95% prediction interval of reflectivity profiles, were used to reliably detect the EZ and ELM peaks (Fig. 2)^47^. This process facilitated both global and spatially resolved rEZR calculations across each B-scan (n = 241) of the SD-OCT raster scan.

To reduce interference from structural changes, retinal areas affected by sub-RPE drusen were automatically excluded from further analysis. These regions were identified as areas where the separation between the RPE and Bruch’s membrane exceeded 15 pixels (~ 100 µm on the image y-axis in high-speed Spectralis OCT imaging). Additionally, regions with MNV or GA, where the absence of EZ or ELM peaks impedes accurate reflectivity assessment, were also excluded.

### Functional probing

As part of the MACUSTAR protocol, BCVA, low-luminance visual acuity, Moorfields Acuity Test, and low-luminance deficit (calculated as low-luminance visual acuity minus BCVA) were assessed using the ETDRS charts and quantified on a logMAR scale. The MAT utilizes pseudo high-pass letter optotypes, designed to reduce low spatial frequency cues, making optotype recognition more dependent on resolution. This improves repeatability and may enhance sensitivity to early AMD-related vision loss^52^. Additionally, contrast sensitivity was assessed using the Pelli Robson Contrast Sensitivity Test, measured on the logCS scale. The assessment of retinal function further comprised mesopic FCP utilizing the MAIA microperimeter (software version 2.5.1, iCare, Padua, Italy). Functional testing procedures have been extensively described in previous publications^23,29^.

Specifically, with regard to FCP, a customized testing grid consisting of 33 stimulus points located at fixed degrees (0°, 1°, 3°, 5°, and 7°), with the fovea serving as the central point, was employed^53^. Mesopic FCP employed a Goldmann size III stimulus (0.43° diameter) for 200 ms, with a dynamic range of 36 dB. A 4–2 strategy adjusted stimulus intensity based on responses to determine thresholds, with a background luminance of 1.27 cd/m^2^. A fixation target (3° radius, 1-pixel thickness) aided in stable fixation. After dilation with 1% tropicamide, participants underwent 5 min of dark adaptation in a fully dark room to ensure consistent testing conditions.

### Analyses of the rEZR and retinal function

Analyses of the rEZR and retinal function employed global, locally averaged, and spatially resolved models. In the global model, the mean rEZR from the entire SD-OCT scan was tested for association with the mesAT in decibels across all 33 stimulus points.

In the locally averaged model, the rEZR was determined at the 33 stimulus points of the FCP grid, assessing areas twice the diameter of each point. The mean rEZR per participant was then tested for its association with the average threshold.

The spatially resolved model calculated the rEZR at each FCP point (twice the stimulus point diameter) to test associations between the rEZR and localized retinal sensitivity (dB), providing a more detailed analysis of structural and functional associations.

The associations between the rEZR and other functional tests were also tested globally and spatially. The global model used the mean rEZR for the entire SD-OCT scan, while the spatially resolved analysis focused on the central subfield (1 mm diameter) of the ETDRS grid to test associations with functional test values.

### Alignment of structural and FCP-derived functional study data

**Fig. 3:**
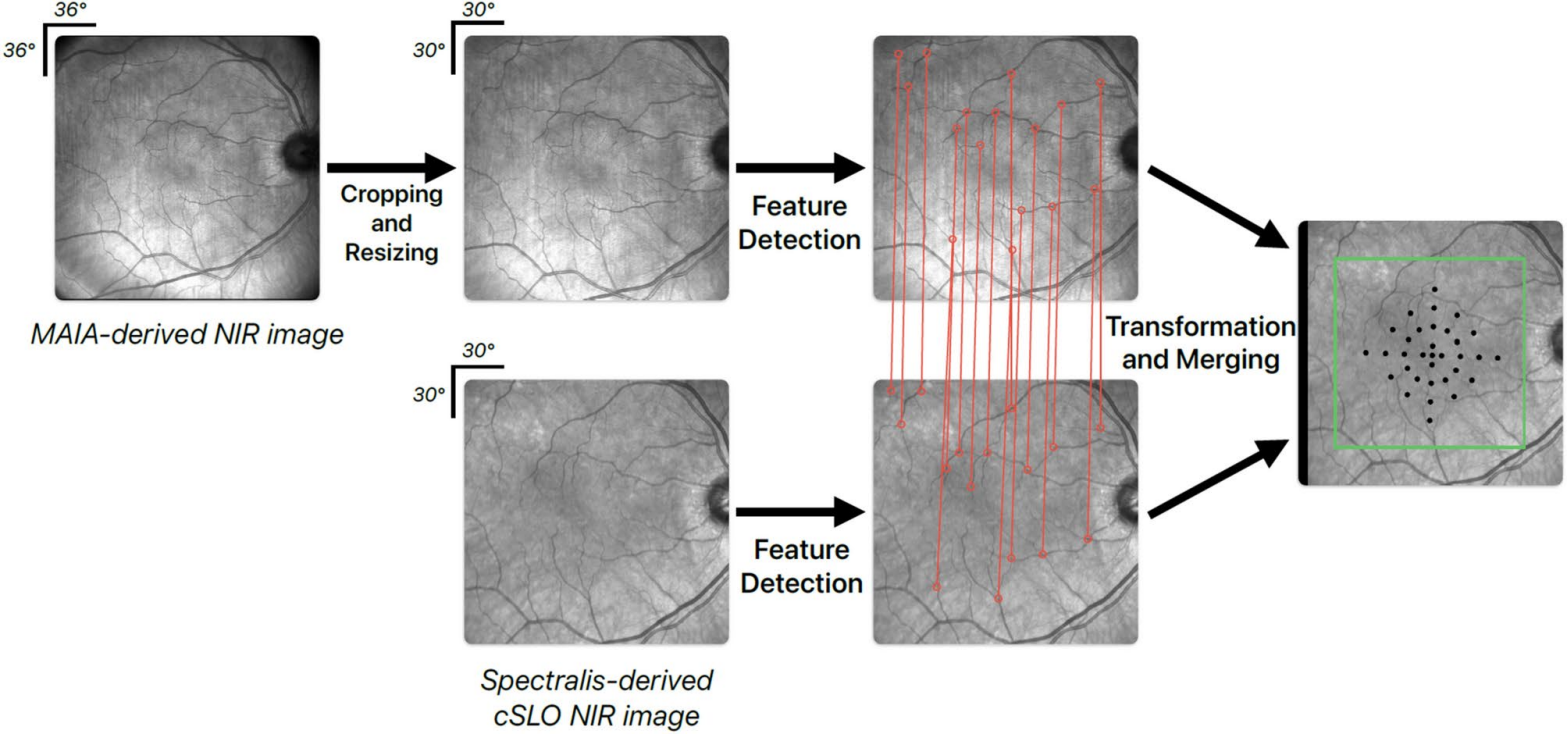
Illustratration of the alignment process of structural, i.e. Spectralis spectral-domain optical coherence tomography (SD-OCT) imaging derived, and functional, i.e. MAIA fundus-controlled perimetry (FCP)-derived, study data. The near-infrared image (NIR) of the FCP-data was cropped and resized to match the image dimensions of the SD-OCT derived NIR image. The deep-learning algorithm “SuperRetina” by Liu et al. was applied to align the NIR images of the FCP and SD-OCT study data using landmark correspondences^54^.

For a spatially-resolved analysis of the rEZR’s functional impact, structural and functional study data were precisely aligned using corresponding en-face near-infrared reflectance images from FCP testing and confocal scanning laser ophthalmoscopy imaging of the SD-OCT dataset (Fig. 3). The initial pre-processing included cropping and resizing the FCP-derived near-infrared reflectance images to match the frame (30° × 30°) and size (768 × 768 pixels) of the confocal scanning laser ophthalmoscopy near-infrared reflectance images. The alignment of the structural and functional data was then accomplished using the “SuperRetina” registration method, a deep learning-based technique specifically trained for the accurate registration of retinal imaging data^54^. This approach allows for a reliable alignment, capable of compensating for noise, artifacts, or variable image quality. The alignment of deep learning-registered images was visually verified by overlaying them in two color channels to ensure accuracy. No deviations were observed, confirming the images were consistently aligned and suitable for analysis.

### Statistical analysis

The study included a descriptive analysis that summarized the baseline characteristics of all participants with valid MAIA exams and available rEZR, focusing on the means and standard deviations.

Analyses were performed using multivariable linear models, adjusted for age, sex, and AMD stage, with functional measures as dependent variables. Additionally, univariate linear regressions were conducted to individually assess these relationships. For the spatially resolved FCP-analysis, a linear mixed-effects model was applied, using the retinal sensitivity values (dB) at each topographically aligned stimulus point as the outcome measures. A patient’s random intercept term was included to account for multiple measurements within the same eye. Further, the spatially resolved model included a spline term for the eccentricity of the rEZR within the volumetric SD-OCT raster scan. For all models, coefficient estimates were assessed, including calculation of 95% confidence intervals.

Descriptive p-values are reported without adjustment for multiple testing, as each functional parameter was analyzed independently for its association with the rEZR in this exploratory study. Since the analyses were not conducted within a single multivariate framework, the risk of false positive findings is minimized, and statistical adjustment for multiple comparisons is not required. A significance level of 0.05 was considered. All analyses were performed using R Version 4.3.0^55^.

## Supplementary Information

Supplementary Information.

## Data Availability

The datasets generated during and/or analyzed during the current study are available from the corresponding author on reasonable request.
