# Supplementary material for: Ellipsoid zone reflectivity as a functional imaging biomarker for age-related macular degeneration: a MACUSTAR study report
Source: Sci Rep. 2025 Jun 20;15:20093. doi: 10.1038/s41598-025-00735-7 (PMC12181357; doi:10.1038/s41598-025-00735-7)
Supplement: Supplementary file 1 — Supplementary Information. [file 41598_2025_735_MOESM1_ESM.docx]

**Supplementary Table 1:** Association of relative ellipsoid zone reflectivity (rEZR) with functional endpoints, including mesopic average threshold (mesAT [dB]), best-corrected visual acuity (BCVA [logMAR]), low-luminance visual acuity (LLVA [logMAR]), and Pelli-Robson contrast sensitivity (PR-CS [logCS]), as assessed in univariate regression models. Associations are reported for global, locally averaged, and spatially resolved analyses. In these models, the control group (no AMD) serves as the reference category.

|  |  | **mesAT [dB]** | | | **mesAT [dB]** | | | **mesAT [dB]** | | | **mesAT [dB]** | | |
| --- | --- | --- | --- | --- | --- | --- | --- | --- | --- | --- | --- | --- | --- |
| global | **Predictors** | **Coefficient Estimate** | **95%-Confidence Interval** | **p-value** | **Coefficient Estimate** | **95%-Confidence Interval** | **p-value** | **Coefficient Estimate** | **95%-Confidence Interval** | **p-value** | **Coefficient Estimate** | **95%-Confidence Interval** | **p-value** |
|  | (Intercept) | 15.1988 | 13.6619 – 16.7357 | <0.0001 | 25.4321 | 24.3230 – 26.5412 | <0.0001 | 42.2384 | 34.6415 – 49.8352 | <0.0001 | 22.4943 | 21.4909 – 23.4976 | <0.0001 |
|  | mean rEZR [AU] | 0.1787 | 0.1416 – 0.2159 | <0.0001 |  |  |  |  |  |  |  |  |  |
|  | AMD stage [early] |  |  | - | -1.4909 | -3.2650 – 0.2833 | 0.0992 | - | - | - | - | - | - |
|  | AMD stage [intermediate] |  |  | - | -2.1419 | -3.4300 – -0.8539 | 0.0012 | - | - | - | - | - | - |
|  | AMD stage [late] |  |  | - | -17.4015 | -19.1454 – -15.6577 | <0.0001 |  |  |  |  |  |  |
|  | age [years] |  |  |  |  |  | - | -0.2877 | -0.3940 – -0.1814 | <0.0001 |  |  |  |
|  | sex [male] |  |  |  |  |  |  |  |  | - | -1.9289 | -3.5845 –-0.2733 | 0.0226 |
| locally averaged | **Predictors** | **Coefficient Estimate** | **95%-Confidence Interval** | **p-value** | **Coefficient Estimate** | **95%-Confidence Interval** | **p-value** | **Coefficient Estimate** | **95%-Confidence Interval** | **p-value** | **Coefficient Estimate** | **95%-Confidence Interval** | **p-value** |
|  | (Intercept) | 16.7412 | 15.4803 – 18.0020 | <0.0001 | 25.4321 | 24.3230 – 26.5412 | <0.0001 | 42.2384 | 34.6415 – 49.8352 | <0.0001 | 22.4943 | 21.4909 – 23.4976 | <0.0001 |
|  | mean rEZR [AU] | 0.1164 | 0.0922 – 0.1406 | <0.0001 |  |  |  |  |  |  |  |  |  |
|  | AMD stage [early] |  |  | - | -1.4909 | -3.2650 – 0.2833 | 0.0992 | - | - | - | - | - | - |
|  | AMD stage [intermediate] |  |  | - | -2.1419 | -3.4300 – -0.8539 | 0.0012 | - | - | - | - | - | - |
|  | AMD stage [late] |  |  | - | -17.4015 | -19.1454 – -15.6577 | <0.0001 |  |  |  |  |  |  |
|  | age [years] |  |  |  |  |  | - | -0.2877 | -0.3940 – -0.1814 | <0.0001 |  |  |  |
|  | sex [male] |  |  |  |  |  |  |  |  | - | -1.9289 | -3.5845 – -0.2733 | 0.0226 |
| spatially resolved | **Predictors** | **Coefficient Estimate** | **95%-Confidence Interval** | **p-value** | **Coefficient Estimate** | **95%-Confidence Interval** | **p-value** | **Coefficient Estimate** | **95%-Confidence Interval** | **p-value** | **Coefficient Estimate** | **95%-Confidence Interval** | **p-value** |
|  | (Intercept) | 22.0366 | 21.1920 – 22.8812 | <0.0001 | 25.8349 | 24.6766 – 26.9932 | <0.0001 | 42.9369 | 35.2613 – 50.6125 | <0.0001 | 22.9658 | 21.9223 – 24.0093 | <0.0001 |
|  | mean rEZR [AU] | 0.0076 | 0.0031 – 0.0122 | 0.0011 | - | - | - | - | - | - | - | - | - |
|  | AMD stage [early] |  |  | - | -1.4927 | -3.2993 – 0.3140 | 0.1054 | - | - | - | - | - | - |
|  | AMD stage [intermediate] |  |  | - | -2.0736 | -3.3858 – -0.7614 | 0.0020 | - | - | - | - | - | - |
|  | AMD stage [late] |  |  | - | -17.5389 | -19.3236 – -15.7543 | <0.0001 |  |  |  |  |  |  |
|  | age [years] |  |  |  |  |  | - | -0.2916 | -0.3990 – -0.1843 | <0.0001 |  |  |  |
|  | sex [male] |  |  |  |  |  |  |  |  | - | -2.0843 | -3.7535 – -0.4151 | 0.0144 |
|  | | | | | | | | | | | | | |

**Supplementary Table 2:** Association of functional tests with relative ellipsoid zone reflectivity (rEZR), AMD stage, age, and sex. Univariate models were fitted separately for each independent variable, with functional tests, including best-corrected visual acuity (BCVA), low-luminance visual acuity (LLVA), Moorfields Acuity Test (MAT), Pelli-Robson contrast sensitivity (PR), and low-luminance deficit (LLD), as the outcome variables. For rEZR, analyses were conducted globally and in the central subfield of the ETDRS grid. Results are presented in individual tables for rEZR (arbitrary units, AU), AMD stage as a categorical variable with the no AMD group as the reference category, age in years, and sex with female sex as the reference category.

|  |  | **BCVA [logMAR]** | | | **LLVA [logMAR]** | | | **MAT [logMAR]** | | | **PR [logCS]** | | | **LLD [logMAR]** | | |
| --- | --- | --- | --- | --- | --- | --- | --- | --- | --- | --- | --- | --- | --- | --- | --- | --- |
| global | **Predictors** | **Coefficient Estimate** | **95%-Confidence Interval** | **p-value** | **Coefficient Estimate** | **95%-Confidence Interval** | **p-value** | **Coefficient Estimate** | **95%-Confidence Interval** | **p-value** | **Coefficient Estimate** | **95%-Confidence Interval** | **p-value** | **Coefficient Estimate** | **95%-Confidence Interval** | **p-value** |
|  | (Intercept) | 0.3606 | 0.2939 – 0.4272 | <0.0001 | 0.5862 | 0.5203 – 0.6520 | <0.0001 | 0.7404 | 0.6831 – 0.7978 | <0.0001 | 1.2645 | 1.2030 – 1.3259 | <0.0001 | -0.2256 | -0.2586 – -0.1925 | <0.0001 |
|  | mean rEZR [AU] | -0.0069 | -0.0085 – -0.0053 | <0.0001 | -0.0077 | -0.0093 – -0.0061 | <0.0001 | -0.0066 | -0.0080 – -0.0052 | <0.0001 | 0.0073 | 0.0058 – 0.0088 | <0.0001 | 0.0008 | 0.0000 -0.0016 | 0.0467 |
| spatially resolved | **Predictors** | **Coefficient Estimate** | **95%-Confidence Interval** | **p-value** | **Coefficient Estimate** | **95%-Confidence Interval** | **p-value** | **Coefficient Estimate** | **95%-Confidence Interval** | **p-value** | **Coefficient Estimate** | **95%-Confidence Interval** | **p-value** | **Coefficient Estimate** | **95%-Confidence Interval** | **p-value** |
|  | (Intercept) | 0.2542 | 0.2030 – 0.3055 | <0.0001 | 0.4827 | 0.4299 – 0.5355 | <0.0001 | 0.6421 | 0.5965 – 0.6877 | <0.0001 | 1.3679 | 1.3187 – 1.4172 | <0.0001 | -0.2284 | -0.2553 – -0.2016 | <0.0001 |
|  | mean rEZR [AU] | -0.0058 | -0.0071 – -0.0043 | <0.0001 | -0.0067 | -0.0082 – -0.0053 | <0.0001 | -0.0054 | -0.0067 – -0.0042 | <0.0001 | 0.0061 | 0.0047 – 0.0075 | <0.0001 | 0.0010 | 0.0003 – 0.0018 | 0.0077 |

|  | **BCVA [logMAR]** | | | **LLVA [logMAR]** | | | **MAT [logMAR]** | | | **PR [logCS]** | | | **LLD [logMAR]** | | |
| --- | --- | --- | --- | --- | --- | --- | --- | --- | --- | --- | --- | --- | --- | --- | --- |
| **Predictors** | **Coefficient Estimate** | **95%-Confidence Interval** | **p-value** | **Coefficient Estimate** | **95%-Confidence Interval** | **p-value** | **Coefficient Estimate** | **95%-Confidence Interval** | **p-value** | **Coefficient Estimate** | **95%-Confidence Interval** | **p-value** | **Coefficient Estimate** | **95%-Confidence Interval** | **p-value** |
| (Intercept) | -0.0404 | -0.0750 – -0.0058 | 0.0224 | 0.1370 | 0.0951 – 0.1789 | <0.0001 | 0.3532 | 0.3148 – 0.3916 | <0.0001 | 1.7085 | 1.6536 – 1.7634 | <0.0001 | -0.1774 | -0.2116 – -0.1432 | <0.0001 |
| AMD stage [early] | 0.0510 | -0.0044 – 0.1063 | 0.0710 | 0.0513 | -0.0157 – 0.1182 | 0.1332 | 0.0650 | 0.0036 – 0.1265 | 0.0382 | -0.0791 | -0.1668 – 0.0087 | 0.0772 | -0.0003 | -0.0550 – 0.0544 | 0.9917 |
| AMD stage [intermediate] | 0.0642 | 0.0240 – 0.1044 | 0.0018 | 0.1000 | 0.0514 – 0.1486 | 0.0001 | 0.0882 | 0.0436 – 0.1329 | 0.0001 | -0.1516 | -0.2153 – -0.0879 | <0.0001 | -0.0358 | -0.0755 – 0.0039 | 0.0771 |
| AMD stage [late] | 0.8032 | 0.7488 – 0.8576 | <0.0001 | 0.7919 | 0.7261 – 0.8578 | <0.0001 | 0.6746 | 0.6142 – 0.7350 | <0.0001 | -0.6210 | -0.7073 – -0.5347 | <0.0001 | 0.0112 | -0.0425 – 0.0650 | 0.6809 |

|  | **BCVA [logMAR]** | | | **LLVA [logMAR]** | | | **MAT [logMAR]** | | | **PR [logCS]** | | | **LLD [logMAR]** | | |
| --- | --- | --- | --- | --- | --- | --- | --- | --- | --- | --- | --- | --- | --- | --- | --- |
| **Predictors** | **Coefficient Estimate** | **95%-Confidence Interval** | **p-value** | **Coefficient Estimate** | **95%-Confidence Interval** | **p-value** | **Coefficient Estimate** | **95%-Confidence Interval** | **p-value** | **Coefficient Estimate** | **95%-Confidence Interval** | **p-value** | **Coefficient Estimate** | **95%-Confidence Interval** | **p-value** |
| (Intercept) | -0.6957 | -1.0189 – -0.3725 | <0.0001 | -0.6430 | -0.9664 – -0.3196 | 0.0001 | -0.3813 | -0.6587 | 0.0073 | 2.3433 | 2.0373- 2.6493 | <0.0001 | -0.527 | -0.2018 – 0.0964 | 0.4866 |
| age [years] | 0.0113 | 0.0068 – 0.0158 | <0.0001 | 0.0133 | 0.0088 – 0.0178 | <0.0001 | 0.0124 | 0.0124 | <0.0001 | -0.0114 | -0.0157 – -0.0071 | <0.0001 | -0.0020 | -0.0041 – 0.0001 | 0.0687 |

|  | **BCVA [logMAR]** | | | **LLVA [logMAR]** | | | **MAT [logMAR]** | | | **PR [logCS]** | | | **LLD [logMAR]** | | |
| --- | --- | --- | --- | --- | --- | --- | --- | --- | --- | --- | --- | --- | --- | --- | --- |
| **Predictors** | **Coefficient Estimate** | **95%-Confidence Interval** | **p-value** | **Coefficient Estimate** | **95%-Confidence Interval** | **p-value** | **Coefficient Estimate** | **95%-Confidence Interval** | **p-value** | **Coefficient Estimate** | **95%-Confidence Interval** | **p-value** | **Coefficient Estimate** | **95%-Confidence Interval** | **p-value** |
| (Intercept) | 0.0903 | 0.0477 – 0.1330 | <0.0001 | 0.2810 | 0.2378 – 0.3243 | <0.0001 | 0.4889 | 0.4512 – 0.5265 | <0.0001 | 1.5454 | 1.5047 – 1.5861 | <0.0001 | -0.1970 | -0.2097 – -0.1717 | <0.0001 |
| sex [male] | 0.0441 | -0.0263 – 0.1145 | 0.2185 | 0.0578 | -0.0136 – 0.1292 | 0.1124 | 0.0258 | -0.0363 – 0.0879 | 0.4139 | -0.0320 | -0.0991 – 0.0351 | 0.3481 | -0.0137 | -0.0450 – 0.0177 | 0.3919 |
